# Fetal-maternal interactions during pregnancy: a ‘three-in-one’ perspective

**DOI:** 10.3389/fimmu.2023.1198430

**Published:** 2023-06-07

**Authors:** Yonghong Zhang, Zhaozhao Liu, Haixiang Sun

**Affiliations:** ^1^ Center for Reproductive Medicine and Obstetrics and Gynecology, Nanjing Drum Tower Hospital, The Affiliated Hospital of Nanjing University Medical School, Nanjing, China; ^2^ Reproduction Center, The Third Affiliated Hospital of ZhengZhou University, ZhengZhou, China

**Keywords:** trophoblast, NK, macrophage, Treg, commensal microbiota, interactions

## Abstract

A successful human pregnancy requires the maternal immune system to recognize and tolerate the semi-allogeneic fetus, allowing for appropriate trophoblasts invasion and protecting the fetus from invading pathogens. Therefore, maternal immunity is critical for the establishment and maintenance of pregnancy, especially at the maternal-fetal interface. Anatomically, the maternal-fetal interface has both maternally- and fetally- derived cells, including fetal originated trophoblasts and maternal derived immune cells and stromal cells. Besides, a commensal microbiota in the uterus was supposed to aid the unique immunity in pregnancy. The appropriate crosstalk between fetal derived and maternal originated cells and uterine microbiota are critical for normal pregnancy. Dysfunctional maternal-fetal interactions might be associated with the development of pregnancy complications. This review elaborates the latest knowledge on the interactions between trophoblasts and decidual immune cells, highlighting their critical roles in maternal-fetal tolerance and pregnancy development. We also characterize the role of commensal bacteria in promoting pregnancy progression. Furthermore, this review may provide new thought on future basic research and the development of clinical applications for pregnancy complications.

## Introduction

A successful human pregnancy requires an intricate balance of maternal tolerance towards the fetus with paternal antigens while providing protection against pathogen invasion (1). This balance is achieved via delicate modulations in the maternal immune system. The placenta is a key mediator that regulates the capabilities of the maternal immune system to maintain the local homeostasis at the implantation site (2). The maternal-fetal interface is considered a complex environment constituted by cells of multiple origins, including fetal originated trophoblasts, and maternal derived decidual stromal cells and decidual immune cells (DICs). The decidual immune cellular part includes several immune cells, including decidual natural killer (dNK) cells, decidual macrophages (DMs), dendritic cells (DCs), and T cells (3). From implantation to delivery, the invading trophoblasts are in close communication with the DICs. Thus, there is an active process of adaptation and modulation from both the maternal and fetal sides, especially at the implantation site. The appropriate communication between the fetal trophoblasts and the maternal DICs contribute to the success of pregnancy.

There is increasing evidence for the presence and importance of ‘commensal’ microbiota in both the pregnant and non-pregnant uterus, although still controversial and not well defined (4, 5). With patten recognition receptors, trophoblasts have the potential to sense and respond to bacterial products (6). The downstream signals may help to induce the tolerogenic immune system, thus allowing embryo implantation and fetus development (7). Furthermore, the interactions between normal bacteria and trophoblasts induce basal expression of type I interferon-β (IFN-β) and downstream interferon stimulating genes (ISGs) in the regulation of maternal immune homeostasis and protection against viral infection (2). Therefore, evidence for a trophoblast-dominated three-way interaction among trophoblasts, decidual immune system, and uterine microbiota is emerging, especially in humans. Exploring the crosstalk at the maternal-fetal interface will help us further understand the development of normal pregnancy and pregnancy complications. Since both human and mice are characterized with highly invasive hemochorial placenta, mice become an important model for investigating human pregnancy mechanism. In the current review, we discuss the crosstalk among trophoblasts, DICs, and commensal microbiota at the maternal-fetal interface in human and mice in order to provide an overview of communication network that promoting normal pregnancy.

## The characteristic and functions (including the modulation on trophoblasts) of decidual immune cells

Decidualization is one of the critical events for a successful pregnancy in humans (8, 9). The decidua provides the embryo with adequate nutritional supply and an immunologically privileged site before placentation (2, 10). Apart from the decidualized stromal cells, the decidua is enriched with multiple immune cells, such as NK cells, macrophages and T cells. DICs jointly contribute to the remodeling of decidua immune homeostasis, subsequently promoting the establishment and maintenance of successful pregnancy. However, dysfunction of DICs is closely associated with pregnancy complications, including implantation failure, recurrent miscarriage (RM), pre-eclampsia (PE), and intrauterine fetal growth restriction (FGR). Therefore, maternal immune homeostasis is the key component for placenta homeostasis (2).

### Decidual NK cells

NK cells, which make up around 70% of immune cells in the uterine endometrium during the secretory phase and early pregnancy, express a variety of activating and inhibitory receptors and cytokines. Human dNK cells are characterized by a unique CD56^bright^CD16^-^ phenotype and encoded several unusual receptors, such as lectin-like receptors, integrin subunits and various killer cell Ig-like receptors (11). Single cell RNA sequence analysis revealed the heterogeneity of dNK cells, namely, dNK1, dNK2 and dNK3 subsets (12). In comparison with the other two subsets, dNK1 cells exhibit higher expression of killer cell immunoglobulin-like receptors (KIRs), leukocyte immunoglobulin-like receptor B1 and human leukocyte antigen (HLA) receptors. Furthermore, the dNK1 population contain enzymes involved in glycolysis and cytoplasmic granule and perforin which protect from pathogen infection. The activating killer cell lectin-like receptors C2 (NKG2C), NKG2E and NKG2A are observed on the dNK2 and dNK1 cells, while dNK2 cells show high expression of X-C motif chemokine ligand 1, which suggests a role in mediating recruitment of other immune cells and trophoblasts at the placenta site (13–15). The smallest proportion, dNK3 cells exhibit high expression of chemokine ligand 5 which suggests a role in modulating invasion of trophoblasts. Although the importance of dNK cells in early pregnancy has been widely acknowledged, it remains an important question whether the dNK phenotypic proportions remain constant throughout pregnancy.

**Figure 1:**
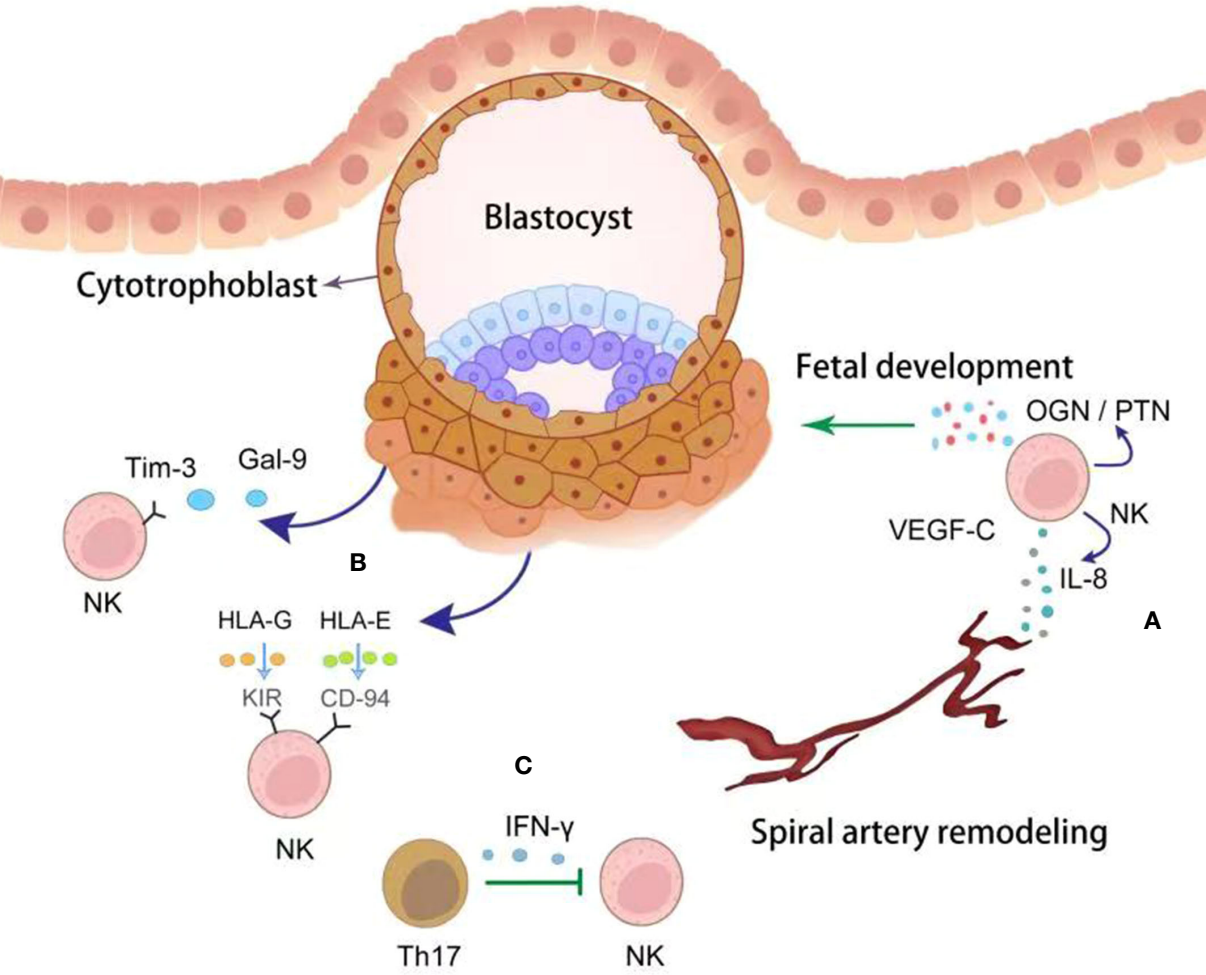
The crosstalk between trophoblasts and dNK cells. NK cells derived cytokines, including VEGF-C, IL-8 and OGN/PTN, promote spiral artery remodeling and fetal development (green point arrow) **(A)**. Correspondingly, trophoblasts educate dNK cells with tolerant phenotype by producing Gal-9 and HLAs (dark blue point arrow) **(B)**. dNK cells were inhibited (green block arrow) by Th17 cells via secreting IFN-γ **(C)**.

Accumulating evidence indicates that dNK cells benefit early pregnancy via several methods (**Figure 1**). Firstly, several studies indicates that dNK cells promote spiral artery (SA) remodeling by producing angiogenic growth factors in early pregnancy, such as interleukin 8 (IL-8), angiopoietin-1/2, and vascular endothelial growth factor (VEGF), initiating SA transformation (16–18). Besides, dNK cells express a wide range of matrix metalloproteinases (MMPs), which can initiate breakdown of the extracellular matrix (19). To prevent excess extravillous trophoblasts (EVT) invasion, dNK cells can also produce cytokines, such as transforming growth factor β (TGF-β), tumor necrosis factor α (TNF-α), and interferon γ (IFN-γ), which inhibit excessive trophoblast invasion in later stages (20, 21). The critical role of uNK cells on SA remodeling was further confirmed in pregnancy complications (18). Reduced dNK numbers have been found in women with FGR and PE, both of which are pathologically characterized by poor SA remodeling and shallow trophoblast invasion (18). The capacity of dNK cells to promote fetal growth and development has been reported. Specifically, CD49a^+^dNK cells have been identified as having the ability to promote fetal development by producing growth-promoting factors, such as pleiotrophin and osteoglycin, before placental establishment (22). Secondly, dNK cells play a role as immunomodulators at the maternal-fetal interface by suppressing inflammatory Th17 cells via IFN-γ to promote maternal immune tolerance (23). Thirdly, dNK cells fight against invading pathogens at the placenta, such as Listeria monocytogenes and Zika virus (ZIKV), via expressing the antimicrobial peptide granulysin (24). Thus, dNK cells play a crucial role in modulating placental vascular remodeling and contribute to the immunomodulatory micro-environment facilitating fetal growth during early pregnancy.

### Decidual macrophages

During reproduction, decidual macrophages (DMs) are present in the decidua, accounting for 20-30% of the DICs. Based on their surface antigens, cytokine profile and functions, DMs could be classified into classically activated (M1, CD14^+^CD86^+^) and alternatively activated (M2, CD14^+^CD206^+^) subtypes, although this was still controversial (25). In order to adapt to different stages of fetal development, the phenotype of DM changes dynamically with the change of microenvironment at different gestational ages. During the embryo implantation window, DMs exhibit M1 phenotype (26). With the implantation and invasion of trophoblasts into the endometrium, DMs change into M1/M2 mixed type, which persists until the first trimester and early stage of the second trimester (26). In the second trimester, DMs polarize towards the M2 phenotype with maternal immune tolerance and promote fetal growth until delivery (27). During delivery, the DMs exhibit M1 phenotype (28). Although accumulating studies has proved the role of DMs, their classification and phenotype remain controversial.

**Figure 2:**
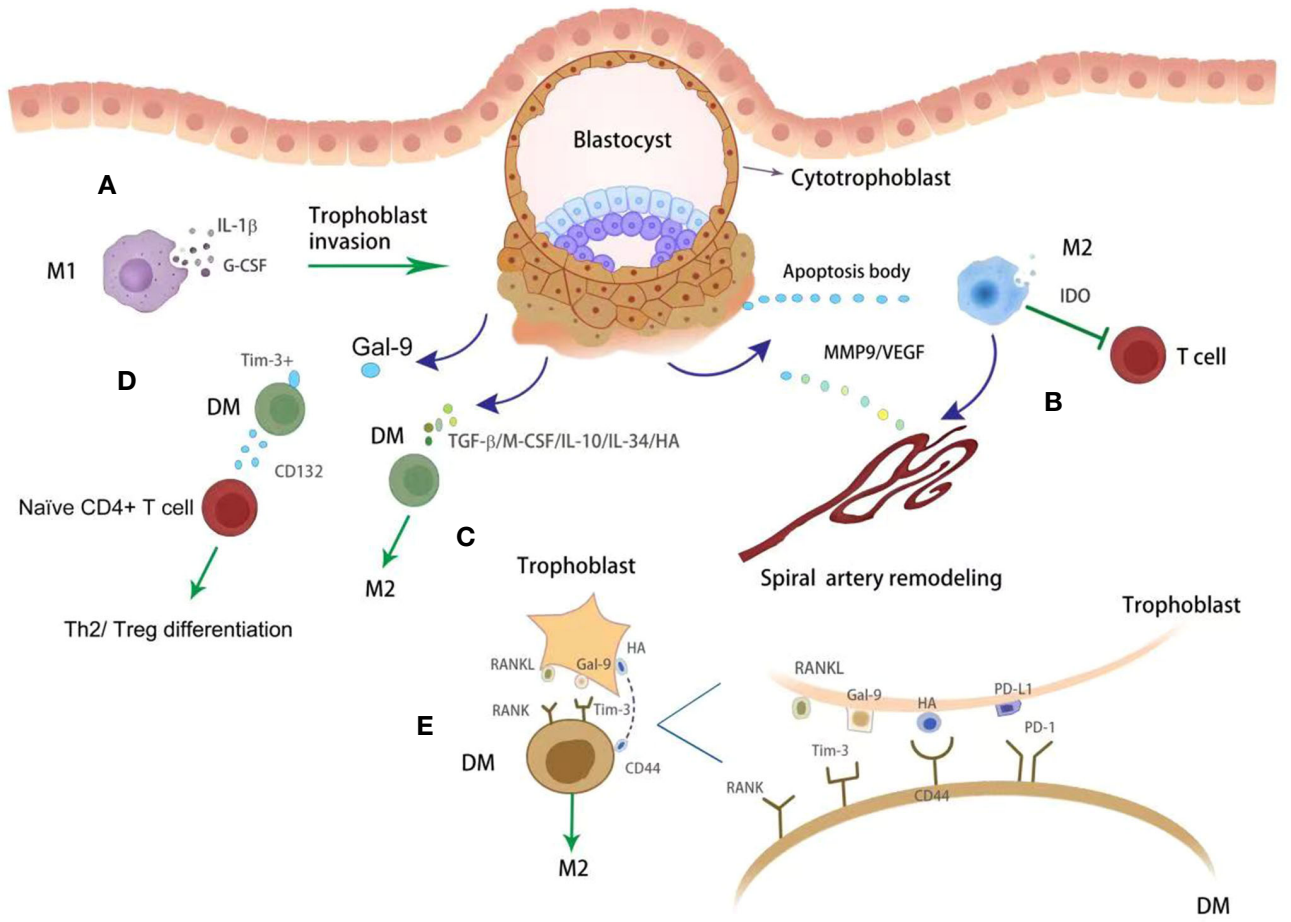
The crosstalk between trophoblasts and DMs. DMs with M1 phenotypes promote trophoblasts invasion *via* releasing IL-1β and G-CSF **(A)**. With phagocytosing apoptotic bodies from placenta, DMs show M2 phenotype which further inhibit T cell activity *via* producing IDO (green block arrow) **(B)**. Apart from immune modulation, DMs also promote artery remodeling by producing MMP9 and VEGF (dark blue point arrow) **(B)**. In addition, DMs were regulated by trophoblasts derived various cytokines (dark blue point arrow), such as TGF-β, M-CSF, IL-10, IL-34 and HA, toward M2 phenotype (green point arrow) **(C)**. The immune checkpoints were also observed functional on modulating DMs immunity. Tim3^+^ DMs promote Th2 and Treg differentiation (green point arrow) *via* producing CD132 **(D)**. Gal-9/PD-L1/RANKL/HA were observed on trophoblasts, which further promote DMs polarization toward M2 phenotype (green point arrow) by binding with specific receptors **(E)**.

DMs play numerous important roles during pregnancy (**Figure 2**). Firstly, DMs are proposed to perform regulatory/homeostatic functions. Through phagocytosis, DMs perform ‘clean up’ apoptotic trophoblasts, preventing activation of pro-inflammatory pathways (29). They also secrete indoleamine 2,3-dioxygenase (IDO) which catabolizes tryptophan and further inhibits T helper cell activation (30). Secondly, DMs perform an antimicrobial role to protect the fetus against infections via expression of pattern recognition receptors, such as CD206, CD209 and CD163 (31). Thirdly, DMs enhance SAs remodeling and trophoblast invasion via producing MMP9 and VEGF, promoting tissue remodeling and angiogenesis (32). It has also been reported that DMs regulate vascular remodeling by secreting placental growth factor and their receptor, FMS-like tyrosine kinase (33, 34). Compared with M1 subsets, M2 macrophages have higher angiogenic potential. M2 macrophages produce a higher level of granulocyte colony stimulating factor (G-CSF) and were able to increase the expression of G-CSFR (35). Macrophage-derived G-CSF promotes the epithelial-to-mesenchymal transition, migration and invasion of trophoblasts via activating PI3K/Akt/Erk1/2 signaling pathway (36). The role of M2 macrophages in implantation was further proved using M2 deleted mice by targeting CD206. In the pregnant M2 deleted mice, lower implantation number and accelerated epithelial cell proliferation, and reduced leukemic inhibitory factor were observed. M2 depleted pregnant mice showed upregulated uterine Wnt/β-catenin signals. Thus, CD206^+^ M2-like macrophages may be essential for embryo implantation through the regulation of endometrial proliferation via Wnt/β-catenin signaling (37). Furthermore, activated DM-derived IL-1β facilitates trophoblast invasion by degrading the extracellular matrix (38, 39). In addition, DM promoted pregnancy via modulating other immune cells. For example, DMs with expression of T cell immunoglobulin domain and mucin domain-3 (Tim-3) induced CD4^+^T cells towards T helper 2 (Th2) and regulatory T (Treg) bias via CD132 (40).

### Decidual T cells

Compared with dNK and DMs, T cells constitute a smaller proportion (10-20%) of DICs, but are important for implantation and placentation. Although multiple adaptive immune cell subsets play a role throughout pregnancy, the key cell mediators are Treg cells and Th cells. At the maternal-fetal interface, approximately 10-30% of CD4^+^T cells are Treg cells, which show immunomodulatory effects on immunity through their signature transcription factor Forkhead box protein 3 (Foxp3) (41, 42). However, effector T (Teff) cells could be classified into different subsets based on their functional phenotypes and cytokine production. For example, Th1 and Th17 cells are pro-inflammatory subsets while Th2 cells are anti-inflammatory during pregnancy (43). Decidual Th1 cells are moderately enriched at the maternal-fetal interface, while the proportions of Th2 and Th17 cells are similar. This signifies the presence of a Th1/Th2/Th17/Treg paradigm in the decidua. Recently, more attention has been paid to the Treg/Th17 balance in the field of reproductive immunity. CD4^+^CD25^+^Foxp3^+^ Treg cells are essential for the anti-inflammatory transition accompanying implantation and placentation. Without the presence of Treg cells, the allogeneic fetuses were uniformly rejected, which was most vulnerable in the pre- and peri-implantation phase (44, 45). Pregnant Rag1(-/-) mice with T cell-deficient are more susceptible to LPS induced PTD, which could be alleviated by adoptive transplantation of Foxp3^+^ cells (46).

**Figure 3:**
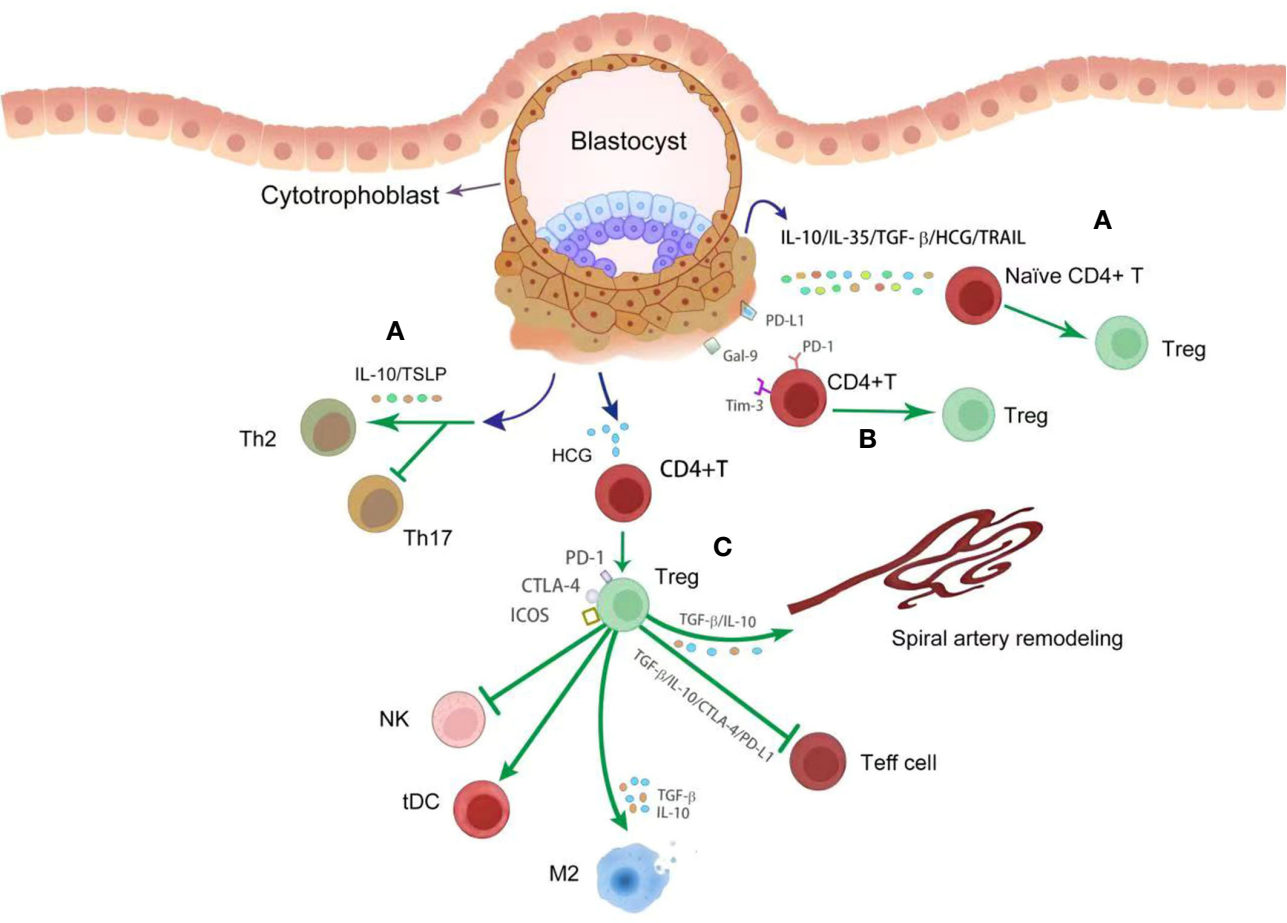
The crosstalk between trophoblasts and decidual T cells. During placentation, trophoblasts derived various cytokines (dark bule point arrow) promote Treg differentiation (green point arrow) and inhibit Th17 response to control the inflammatory response **(A)**. These cytokines include IL-10, IL-35, TGF-β, HCG, TSLP and TRAIL. With engagement with Gal-9 and PD-L1 from trophoblasts, Treg differentiation (green point arrow) was enhanced **(B)**. By producing anti-inflammatory cytokines and immune modulatory proteins, such as TGF-β, IL-10, PD-L1 and CTLA-4, Treg cells profoundly inhibit Teff cell response and NK activation (green block arrow), and promote the differentiation of tDC and M2 macrophages (green point arrow) **(C)**. In addition, Treg cells promote spiral artery remodeling (green point arrow) via TGF-β and IL-10 **(C)**.

Treg cells facilitate implantation and placental development via at least three mechanisms (**Figure 3**). Firstly, Treg cells have a profoundly inhibitory effects on effector CD4^+^T and CD8^+^T cells (47, 48). Uncontrolled Teff cells had adverse effects on placental development in an antigen-independent manner, possibly through the release of inflammatory cytokine and antigen-dependent trophoblast cytotoxicity (49). Decidual Treg cells secret TGF-β and IL-10 and express CD25, cytotoxic T lymphocyte-associated antigen-4 (CTLA-4), and programmed cell death ligand 1 (PD-L1), all of which are signature mediators of Treg inhibition and may contribute to Teff cell constraint in early pregnancy (43, 50–52). Secondly, Treg cells regulate other immune cells in the decidual environment, facilitating anti-inflammatory and tolerogenic phenotypes in M2 macrophages and tolerogenic DCs via TGF-β and IL-10, and CTLA-4. Treg cells may be important modulators for the phenotype and function of NK cells during implantation by controlling the release of IL-15 release from DCs and inhibiting NK cytolytic activity (53). These coordinated interactions enable Treg cells to control the inflammatory response and oxidative stress associated with trophoblast invasion. Thirdly, Treg cells are a critical modulator of SA remodeling, which is essential for normal placental development. Treg-deficient pregnant mice exhibit consistent damages in uterine SA modification, decreased placental blood flow and FGR (54, 55). Acute depletion of Foxp3^+^ Treg cells in first trimester of pregnancy leads to uterine artery dysfunction in later pregnancy, which is closely associated with increased production of vasoconstrictor endothelin-1 (55). Poor trophoblast invasion and SA transformation failure were also seen in mice after depletion of Treg cells (56). Therefore, Treg action is indispensable for successful pregnancy.

Apart from modulatory effects, Treg cells show significant phenotypic plasticity, as they can transdifferentiate into potentially deleterous Th17 cells in response to certain environmental signals. This was also observed in pre-eclamptic women in the third trimester (51). The ability of Treg cells to transition between pro- and anti-inflammatory cell types provides a therapeutic opportunity for inflammatory pregnancy complications. PD-L1 was one of the regulators promoting Th17 cells or Foxp3^+^IL-17^+^ cells transdifferentiated into Treg cells, providing evidence supporting the hypothesis that PD-1 might be an ideal therapeutic target in pregnancy complications (51).

## The commensal microbiota

Although still controversial and not clearly defined, there are a growing number of studies proving the evidence of the presence and importance of a commensal microbiome in both the pregnant and non-pregnant uterus (4). Human placenta has a unique microbial niche, which is composed of nonpathogenic symbiotic microbiota from the Firmicutes, Tenericutes, Proteobacteria, Bacteroidetes, and Fusobacteria phyla (5). Overall, the placental microbiome is most similar with the human oral microbiome (5, 57). *In vivo* studies have shown that the normal maternal microbiota may help induce immune tolerance, allowing receptivity and preventing rejection of the fetal-placental unit (7). However, abnormal maternal microbiota might be associated with pregnancy loss. The increase in *Ureaplasma* species in uterine endometrium microbiota of women with recurrent pregnancy loss, were risks of miscarriage and preterm delivery in subsequent pregnancies with normal chromosome karyotype (58). During peri-implantation phase, the presence of *Lactobacillus*-dominated endometrium microbiota (>90% *Lactobacillus* spp.) was associated with significantly increased implantation, pregnancy, and live birth rates compared with those with non-*Lactobacillus*-dominated microbiota (<90% *Lactobacillus* spp. with >10% of other bacteria) in endometrium (59). Pathogenic bacteria, such as *Streptococcus, Staphylococcus, Neisseria, and Klebsiella* were most frequently observed in the endometrium of patients with repeated implantation failure (60). Thus, the maternal endometrium microbiota is closely associated with pregnancy development. Keeping endometrium microbiota homeostasis might contribute to embryo implantation and pregnancy development.

## Immune cells and trophoblasts interactions

Trophoblast invasion and vascular remodeling are the two most crucial events during placental development. During early pregnancy, decidual immune cells showing pro-inflammatory profile were closely associated with placentation, by releasing soluble cytokines and cell-to-cell dependent interactions to facilitate trophoblast invasion and vascular remodeling (61). Reduced trophoblast invasion and vascular transformation can result in poor placental perfusion, potentially leading to pregnancy disorders, such as recurrent miscarriage (RM), FGR and PE. To accept the semi-allogenic fetus, the maternal immune system will establish an immunological tolerance pre-dominated state, while preserving immune response against infection. Interestingly, the shift of immune condition from pro-inflammatory to immune tolerance along with implantation and placentation is modulated by trophoblasts of fetal origin. Therefore, the fetus might be not passively accepted by his mother, but actively educates maternal immune cells switch to anti-inflammatory profile. However, excessive immune response is considered the leading cause of the unexplained pregnancy loss (62). Since the modulation of DICs on trophoblasts have been showed in the former section, we only review the modulatory effects of trophoblasts on DICs.

### The modulation of trophoblasts on dNK cells

Trophoblast is the only cell type at the maternal-fetal interface that carries paternal antigens and express a unique repertoire of MHC ligands. dNK cells are modulated by trophoblasts (**Figure 1**). HLA-C, -E, and -G are MHC ligands expressed by EVT, being the targets of dNK cells. As we have mentioned, dNK cells display high level of the inhibitory receptors KIRs, which engage with HLA-G to inhibit NK cytotoxicity (63). Another non-classical HLA class I molecule, HLA-E, is also expressed by trophoblasts and regulate dNK cytotoxicity through direct interaction with the inhibitory receptors CD94/NKG2A (64, 65). Asymmetric growth restriction and abnormal brain development were observed in NKG2A-deficient mice (65). The non-functional HLA-B→HLA-E→NKG2A pathway exposes women to greater pre-eclampsia risk (65). Additionally, HLA-C attenuates NK cell cytotoxicity by interacting with KIRs expressed on NK cells (66). At the maternal-fetal interface, the trophoblast-derived key metabolic enzyme, IDO, degrades tryptophan and has been shown to reduce the cytotoxicity of dNK-cells by downregulating expression of NKp46 and NKG2D, as well as the cytotoxicity of peripheral NK cells (67). These findings demonstrate the important role of IDO in the maintenance of a normal pregnancy (67). Trophoblasts autophagy inhibits the killing capacity of NK cell through insulin growth factor (IGF)-2 (68). Low level of trophoblastic autophagy leads to the increased production of IGF-2, which in turn leads to the high cytotoxicity of NK cells attacking the normal cells in RM patients (68). As a recently defined immune checkpoint, Tim-3 exhibits modulatory effects on T cell immunity (67). Interestingly, Tim-3 was also detected on dNK cells (69). By engagement with Tim-3, galectin-9 (Gal-9) produced by human trophoblast induce the transformation of peripheral NK cells into a dNK like phenotype (70). Furthermore, after ligation with Gal-9 on trophoblasts, Tim-3^+^dNK cells exhibited inhibited cytotoxicity due to decreased production of perforin in comparison with Tim-3^-^ dNK cells (70). Therefore, further exploration on the mechanisms that modulate the functions and phenotype of dNK cells during pregnancy may contribute to the development of novel treatments to prevent pregnancy failure.

### The crosstalk between trophoblasts and DM in shifting towards M2 phenotype

DMs play an active role in several events throughout pregnancy, including embryo implantation, placentation, fetus development, and parturition. CD14^+^ DM were observed closely to the EVTs. Correspondingly, DMs are also regulated by trophoblasts (**Figure 2**). Trophoblasts secreted soluble factors, such as TGF-β, macrophage colony stimulating factor and IL-10 (71). These factors induce the differentiation of monocytes into M2-like macrophages, which are characterized by the expression of CD163, CD206 and CD209, and are known for their enhanced phagocytic capacity (72, 73). Furthermore, trophoblasts derived IL-34 polarizes macrophages into a decidual-like phenotype *in vitro* (74). Pregnancy-specific glycoprotein (PSG) are released by the placenta and are the most abundant fetal proteins in the maternal blood at the end of pregnancy (75). The expression of VEGF was upregulated by PSGs both in RAW 264.7 cell line and in human peripheral monocytes derived macrophages (76). These findings suggest that placenta derived PSGs may play important roles in vascular modification by regulating macrophages. In addition to cytokines and chemokines, trophoblasts secret hyaluronic acid, the most abundant component of the extracellular matrix in all mammalian tissues (77), which can induce M2 polarization of macrophages at the maternal-fetal interface by interacting with CD44 and activating the downstream PI3K/AKT-STAT-3/STAT-6 signal pathway (77).

Tim-3 is constitutively expressed on macrophages (78), and Gal-9, was observed on trophoblasts during pregnancy (79). The ligation of Tim-3 and Gal-9 has been shown to modulate macrophage function at the maternal-fetal interface (80). Blocking Tim-3 signaling by Tim-3 blocking antibodies in pregnant mice inhibits the phagocytic potential of uterine macrophages resulting in a building up of apoptotic bodies at the uteroplacental interface that elicits a local immune response (81). Another immune checkpoint PD-1/PD-L1 axis also plays a crucial role in modulating immune cell homeostasis. PD-L1 was observed on the trophoblasts, while decreased expression was confirmed in women with pregnancy complications such as RM (25). The engagement of PD-L1 and PD-1 promoted macrophages polarization towards M2 phenotype (82). HLA-G was highly expressed in EVTs and its soluble form can reduce the expression of CD86 and increase the expression of CD163 in macrophages (83). Soluble HLA-G5 polarized macrophages increased the production of IL-6 and C-X-C motif ligand 1, which further enhance trophoblast invasion (83). Receptor activator of nuclear factor κB (NF-κB) ligand (RANKL) and its TNF-family receptor RANK are involved in multiple activities. With the expression and secretion of RANKL, trophoblasts could promote macrophage different into M2 subtype via AKT/STAT6-Jmjd3/IRF4 signaling pathway (84). However, low levels of RANKL in trophoblasts and RANK on DMs was observed in women with miscarriage (84). This was further demonstrated by using RANKL^-^/^-^ mice, which exhibited macrophage dysfunction and increased fetal loss (84). However, adoptive transfer of RANK^+^ macrophages alleviate the fetal loss induced by macrophage depletion in mice (84). Metabolic immunity is a hot topic in tumor macrophage immunity. The research on DM and metabolism is emerging. Lactic acid (LA) is produced from glucose through glycolysis. Gao et al. (85) found that trophoblast-derived LA orchestrates DM polarization through SRC/LDHA signaling in human early pregnancy. Under normoxic and anoxic conditions, M2 or M1 macrophage polarization is triggered by LA via oxidative phosphorylation and glycolysis, respectively. Blocking the intake of LA could improve pregnancy outcome in the animal model, indicating that it is a potential therapeutic target for pregnancy loss in the future (85). However, the association between amino acid and lipid metabolism and DM polarization is still unknown.

In recent years, extracellular vesicles (EVs) have been shown to be one critical medium in maternal-fetal communication (86). EVs include several types, such as micro-vesicles, exosomes and apoptotic bodies, which could be released by trophoblasts and macrophages (86, 87). The concentration of EVs in peripheral blood is approximately 5-fold higher in pregnant women compared with the non-pregnant women (88). The exosomes produced by placental trophoblasts modulate monocytes to produce G-CSF, IL-1β, IL-6 and TNF-α, which further contribute to embryo implantation, stromal remodeling and angiogenesis (89). DM-derived EVs deliver miR-153-3p, miR-146a-5p and mi-R146b-5p inhibiting proliferation, migration and invasion of trophoblasts, associated with the development of unexplained RM (90, 91). However, more studies are required to further clarify the role of EVs during human pregnancy.

### The crosstalk between trophoblasts and T cells in promoting Treg function

At the implantation site, placental trophoblasts are considered to be ‘foreign cells’ with paternal antigen (92) invading deeply and actively modulating T cell homeostasis (93). Emerging evidence proves that trophoblasts can induce Treg tolerance by producing various immune mediators, including chemokines and cytokines, hormones, EVs and immune checkpoint ligands (**Figure 3**). Trophoblast cells also promote T cell immunity toward Th2 bias and inhibit Th17 cells through producing cytokines such as IL-10 and thymic stromal lymphopoietins (94). Shifting Th1/Th2/Th17/Treg paradigm toward Treg dominance at the implantation site is critical for establishing a tolerant microenvironment (95). Several studies found that trophoblast-derived cytokines, such as IL-10, IL-35, TGF-β, HCG and TNF-related apoptosis-inducing ligand, enhanced Foxp3 expression in naive CD4^+^T cells and increased the production of Treg specific cytokines (73, 96–99). Furthermore, trophoblasts derived HCG promoted Treg differentiation and the expression of immune checkpoints, including PD-1 and CTLA-4, and transdifferentiate Th17 cells into Th subsets with an anti-inflammatory profile (99). Immune checkpoint molecules are coinhibitory receptors observed on surface of T cells, like PD-1, Tim-3 and CTLA-4 (79). One of the most widely explored is the PD-1/PD-L1 axis in human pregnancy (79). PD-L1 is present in the placenta throughout gestation (79). The engagement of PD-1 and PD-L1 leads to decreased production of proinflammatory cytokines from activated CD4^+^T cells when cocultured with trophoblasts (100). PD-1 was an important mediator in Treg-induced fetal protection in the CBA/J×DBA/2 murine model (101). Blocking PD-L1 via PD-L1 mAb administration in animal model significantly increases fetal loss, which were associated with Treg deficiency at the maternal-fetal interface (102). Furthermore, trophoblasts originated IL-27 and Gal-9 activate the Tim-3 signaling pathway in CD4^+^T cells and Treg cells, thus promoting accumulation of Tim-3^+^Treg cells (103). The abnormal expression of IL-27 and Gal-9 is associated with decreased Treg proportion and impaired immune tolerance in pregnancy loss patients (103). The findings on immune checkpoint molecules modulating T cell immunity during pregnancy indicate their potential for immunotherapy.

## Commensal bacteria and trophoblasts interactions

Trophoblasts constitutively express pattern recognition receptors, including toll like receptor (TLRs) and NOD-like receptors, which can recognize damage associated molecular patterns that are produced by damaged tissues and dying cells (104, 105), and pathogen-associated molecular pattens from bacteria, viruses and other microorganisms (106–109). Instead of inducing the classical NF-κB mediated inflammatory response, lipopolysaccharide (LPS) engagement with TLR4 in trophoblast cells resulted in the production of type 1 IFNs via the MyD88-independent pathway (110). The interaction of LPS and TLR4 leads to the phosphorylation of tank-binding kinase (TBK) and interferon regulatory factor 3 (IRF3), inducing IFN-β expression in trophoblasts (110). Therefore, these findings highlight the potential role of endometrium normal microbiota on maintaining constitutive expression of IFN-β via the TLR4/TBK3/IRF3 signaling in trophoblasts.

As one of the IFN family, IFN-β is capable of inducing an anti-microbial response, modulating innate immunity, and inducing the activation of the adaptive immune system (111). By binding with IFNARs, basal IFN-β promotes the expression of ISGs with immune modulatory activation, as well as provide immune protection against invading pathogens (2). Therefore, the basal IFN-β from trophoblasts is critical for immune homeostasis and preventing virus towards the fetus. However, the effective of IFN-β is dose dependent. Excessive IFN-β responses have harmful effects and facilitate the development of autoimmune diseases (112). Therefore, proper regulation of the expression and activation of type I IFNs is essential for tissue homeostasis (2).

During early pregnancy, trophoblasts produce multiple ISGs in the presence of IFN-β (110). These ISGs regulate immune cell functions and promote immune homeostasis at the implantation site by inducing tolerance to paternal antigens and protecting against pathogen infections. One of the key roles of the placenta is to prevent vertical transmission of the virus, which may be fetal to the fetus and may also lead to serious developmental problems (2). With the infection of ZIKV, trophoblasts can strongly induce IFN-β signaling, which in turn results in the production of several ISGs against virus (110), such as ISG20. The recent publication proved that ISG20 is critical for protecting placenta against ZIKV replication (113). The essential role of IFN-β and ISGs in virus defense was further demonstrated by *ifnar*-/- pregnant mice, which showed increased viral titers in *ifnar*-/- placenta and fetus when challenged by the viruses (such as ZIKV, MHV68 and HSV-2) (114). When challenged with LPS, wild-type mice exhibited normal pregnancy outcomes. In contrast, IFN receptor-deficient mice were more sensitive to LPS and experienced preterm birth within 24 h. IFN receptor deficiency may lead to increased production of pro-inflammatory cytokines, such as TNF-α, IL-6 and IL-8, and the development of preterm birth (115). Thus, the deficiency of IFN-β signaling resulted in uncontrolled placental-fetal viral infection and maternal mortality.

During pregnancy, ISGs are critical not only on antiviral infection, but also on modulating immune cell function (116). Trophoblasts modulate macrophage polarization by producing ISGs, such as PD-L1 (82). Trophoblast-derived PD-L1, which is modulated by IFN-β, contributes to macrophage polarization towards M2 phenotype (56). Nicotinamide phosphoribosyltransferase, another trophoblast-derived ISGs, is known as a critical modulator of macrophages polarization and migration (117). Despite previous research, the functions of ISGs on modulating immune cell functions during pregnancy remains unclear and requires further exploration.

## Commensal microbiota and DICs interactions

The crosstalk between DICs and commensal microbiota has rarely been reported. Commensal bacteria might contribute to homeostasis by modulating the innate and adaptive immune system in the endometrium (118). Since classical antigen presenting cells (APCs) are abundantly in the endometrium, such as macrophages and DCs, these cells should have close interactions with the commensal bacteria. Their communications might subsequently promote pregnancy development by two methods: a, macrophages and DCs promote commensal microbiota homeostasis; b, endometrium bacteria could be recognized by macrophages and DCs and further induce adaptive immune tolerance towards the fetus. Further, the downstream effects of triggering the immune system at the maternal-fetal interface by bacteria is the activation of uterine NK cells and the development of specific subsets of T-cells, characterized by high number of Treg cells and lower number of Th17 cells (118). How the microbiota-immune cells interactions affect the maternal immune response during implantation and pregnancy is not clearly understood.

## Immunotherapy of RM

The harmonious crosstalk among fetal derived trophoblasts, maternal derived DICs, and the commensal microbiota contribute to the maternal-fetal immune tolerance. Although the interactions between DICs and commensal microbiota has rarely been reported. Their crosstalk should contribute to the establishment of maternal tolerance and pregnancy development. Abnormal dialog between the mother and fetus can lead to a deficiency of maternal tolerance and subsequently result in pregnancy failure. Notably, RM is one of the big challenges in reproductive medicine and placed a heavy burden on patients and their families (119). Therefore, effective therapies are urgently required.

The maternal-fetal interactions during pregnancy have significant clinical implications for the therapy of RM. In the past decades, various immunotherapies have emerged, including lymphocyte immunotherapy, intravenous immunoglobulin therapy, intralipid and TNF-α inhibitor (120–123). However, the effectiveness of the current immunotherapies is still controversial. All the novel therapies should be processed based on clear evidence of pregnancy development mechanisms. Until now, great efforts had been done trying to characterize the alloimmune immunity at the maternal-fetal interface. And some new targets had been proposed, such as PD-1, Tim-3 and IFN-β. PD-1 and Tim-3 are immune checkpoint inhibitors (ICIs), the effective therapeutic strategy to restore antitumor immunity by targeting immune checkpoint molecules (124). ICI strategies have shown remarkable success in clinical studies of several malignant tumors (125, 126). Administration of agonists to these ICIs might prevent the development and improve the outcome of pregnancy complications. However, the reproductive safety must be carefully considered. Fundamentally, it is critical to focus on the interactions among trophoblasts, DICs, and microbiota during pregnancy to reveal the mechanisms of normal pregnancy and pave the way for the promising immunotherapy of RM.

## Conclusion and perspectives

The crosstalk among DICs, trophoblasts, and commensal microbiota are critical to ensuring maternal immune tolerance to the semi-allogenic fetus and effective immune protection against pathogens, ultimately safeguarding the smooth progression of pregnancy until successful delivery, especially in the highly invasive hemochorial placenta as seen in humans and mice. However, abnormal conversation among trophoblasts, DICs and microbiota might directly lead to the development of pregnancy complications, such as RM. Therefore, further exploration on the three-way interaction among trophoblasts, decidual immune system, and uterine microbiota will provide fundamental scientific basis for the development of immunotherapies for pregnancy complications. Although efforts had been done, numerous questions remain: (і) Since trophoblasts, DICs, and endometrium microbiota are communicated with each other, which one plays the dominant role? (ii) As pregnancy is characterized by multiple stages, which crosstalk plays a fundamental role at specific stages? (iii) Can microbiota be developed to modulate maternal-fetal tolerance?

## Author contributions

YZ wrote and revised the manuscript. ZL wrote and designed the figures. HS revised the manuscript. All authors contributed to the article and approved the submitted version.
