# Corrigendum: Fetal-maternal interactions during pregnancy: a ‘three-in-one’ perspective

**DOI:** 10.3389/fimmu.2023.1249476

**Published:** 2023-07-18

**Authors:** Yonghong Zhang, Zhaozhao Liu, Haixiang Sun

**Affiliations:** ^1^Center for Reproductive Medicine and Obstetrics and Gynecology, Nanjing Drum Tower Hospital, The Affiliated Hospital of Nanjing University Medical School, Nanjing, China; ^2^Reproduction Center, The Third Affiliated Hospital of ZhengZhou University, ZhengZhou, China

**Keywords:** trophoblast, NK, macrophage, Treg, commensal microbiota, interactions

In the published article, there was an error in affiliation 1. Instead of “Center for Reproductive Medicine and Obstetrics and Gynecology, Drum Tower Hospital, Nanjing University Medical School, Nanjing, China”, it should be “Center for Reproductive Medicine and Obstetrics and Gynecology, Nanjing Drum Tower Hospital, The Affiliated Hospital of Nanjing University Medical School, Nanjing, China”.

In the published article, there was an error in the Funding statement. “Jiangsu Natural Science Funds” should be written as “Natural Science Foundation of Jiangsu Province”. The correct Funding statement appears below.

The authors apologize for these errors and state that they do not change the scientific conclusions of the article in any way. The original article has been updated.

